# Deep Investigation Into the Electrodeposition Model of Mg Battery Anode

**DOI:** 10.3389/fchem.2022.940559

**Published:** 2022-06-13

**Authors:** Yingmei Zhou, Zhengnan Wei, Jing Xu, Changguo Chen

**Affiliations:** ^1^ School of Chemical Engineering, Shandong Institute of Petroleum and Chemical Technology, Dongying, China; ^2^ Postdoctor Scientific Research Station of Shengli Petroleun Administration, SINOPEC, Dongying, China; ^3^ College of Chemistry and Chemical Engineering, Chongqing University, Chongqing, China

**Keywords:** crystal growth, deposition, metals and alloy, electrocrystallization mechanism, instantaneous nucleation

## Abstract

Analysis of nucleation/growth dynamics is important to understand the molecular mechanism on the electrode surface. The electrocrystallization mechanism of Mg anode in aqueous electrolyte was comprehensively investigated which can help us understand the surface discharge mechanism of Mg anode and provide a new theoretical idea for the development of high performance magnesium ion battery. The influence of applied potential signals on normal growth constant and active site numbers was studied using *i*-*t* transient curves. The dimensionless processed transient curves confirmed that the initial nucleation/growth process of Mg electrode in aqueous solution followed the diffusion-controlled three-dimensional instantaneous nucleation model.

## 1 Introduction

Energy storage and conversion are strongly associated with our daily life (Cui et al., 2015; Cui et al., 2017; Ke et al., 2021). Mg ion batteries (MIBs) are popular owing to their low toxicity, abundant resources, and high theoretical volumetric capacity (Shuai et al., 2020; Xu et al., 2020). However, the development of MIBs lags behind that of Lithium ion battery (LIB) because of intrinsic problems of self-corrosion, negative difference effects, and delayed action (Xu et al., 2019). These problems are caused by passive film layers that spontaneously form on electrode surface in aqueous solution. Thus, the process of crystal growth of metal Mg in aqueous solution at the initial electrodeposition stage should be investigated (Morisue et al., 2003). This process involves crystal nucleus formation and crystal growth, both of which play a decisive role in determining the microstructure of electrodeposition products (Garland et al., 2001; Berthier et al., 2004; Kelber et al., 2006; Radisic et al., 2006). In recent years, various electrochemical techniques have been used to study the effect of different factors on the crystal growth mechanism of electrode surface. The electrochemical nucleation and crystal growth mechanisms of metal materials can be determined by comparing time–current transient curves with typical theoretical models.

As one of the most representative topics in electrochemistry knowledge system, electrodeposition process has been attracting much attention of both scientists and engineers. A typical electrodeposition process refers to metal electrodeposition at the electrode/electrolyte interface under the action of an electric field. Crystal nucleation and growth rate determine crystal size. When nucleation rate is higher than growth rate, films with a relatively dense surface can be obtained and vice versa (Heerman and Tarallo, 2000; Ma et al., 2015). The electrodeposition theory evolved from the Butler–Volmer equation, which mainly discusses the relationship between the electric potential (η) and the surface current density of metal substrates (Piatti et al., 1969; Budevski et al., 2000). Four types of electrocrystallization models are presently known: two-dimensional instantaneous nucleation, two-dimensional continuous nucleation, three-dimensional instantaneous nucleation, and three-dimensional continuous nucleation (Bewick et al., 1962).

Nucleation is a crucial step in the metal electrodeposition process. The competition between nucleation rate and crystal growth rate determines the grain size that forms. Small grains can be obtained at high nucleation rates (Armstrong and Fleischmann, 1982; Xu et al., 2021). By comparison, crystal growth rate influences the microstructure of deposited metals, for example, the deposition morphology of fiber structures obtained at high crystal growth rates. Therefore, the mechanism of metal nucleation and its corresponding kinetic process at different electrochemical conditions should be explored.

Analysis of nucleation/growth dynamics is important to understand the molecular mechanism on the electrode surface. In this paper, the electrocrystallization mechanism of Mg anode in aqueous solution at the initial electrodeposition stage was comprehensively investigated which can help us further understand the surface discharge mechanism of Mg anode and provide a new theoretical idea for the development of high performance magnesium ion battery.

## 2 Experimental

All chemicals were of analytical grade. Magnesium nitrate hexahydrate (Mg(NO_3_)_2_^**.**^6H_2_O, 99.0%), Magnesium sulfate anhydrous (MgSO_4_, 99.0%), AZ31B Mg alloy was purchased from Ao-Xin Anticorrosion Materials Co., Ltd. (Jiaozuo, China). The chemical composition of the alloy was (wt %) 3% Al, 1% Zn, 0.2% Mn, and balance Mg. The dimensions of the working electrodes were (1 × 1 × 0.6 cm) linked with copper wire and embedded in epoxy resin used in the experiments, with exposed surface area of 1 cm^2^. The electrolyte composition was MgSO_4_-Mg(NO_3_)_2_ (0.14 mol/L MgSO_4_, 1.86 mol/L Mg(NO_3_)_2_) (Xu et al., 2019; Xu et al., 2020).

Electrochemical measurements were conducted using a CHI 660E electrochemical workstation at room temperature.

## 3 Results and Discussion

### 3.1 Determination of Nucleation Model

#### 3.1.1 I-t Transient Current Curves of Mg Electrodes

**FIGURE 1:**
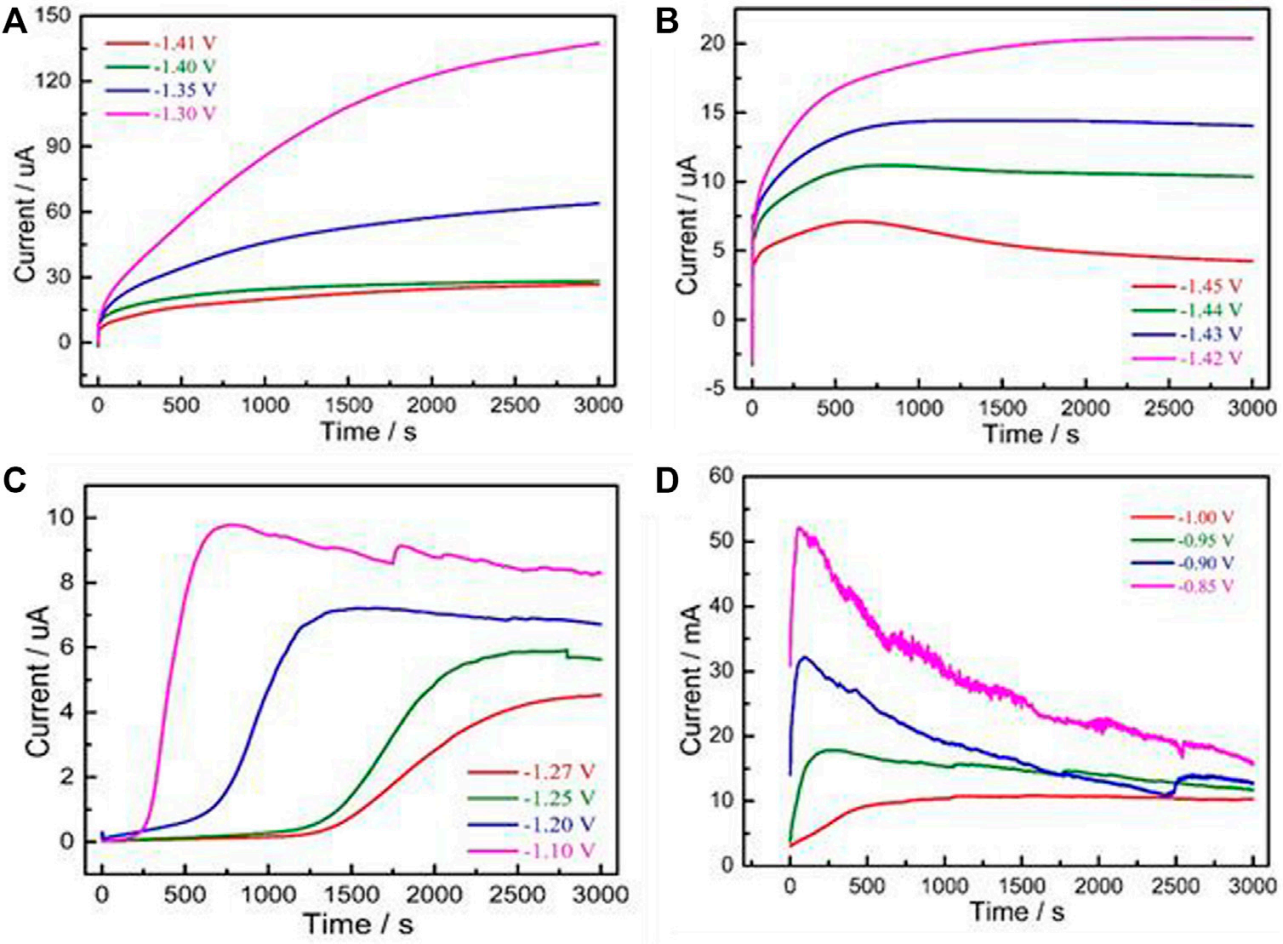
I-T transient net current curves of Mg electrode: **(A)** −1.30∼−1.41 V; **(B)** −1.42∼−1.45 V; **(C)** −1.10∼−1.27 V; **(D)** −0.85∼−1.00 V.

The current–time curves of Mg electrode in aqueous electrolyte are shown in Figure 1. The transient current–time curves of Mg electrode in aqueous solution at different testing voltages (−1.45 to −1.42 V) are plotted in Figure 1A. The common feature was that the response current rapidly increased to maximum and formed the first peak current. This current was related to the double layer charging process, which was caused by the formation of crystal nucleus and the growth of a new phase.

The transient current–time curves of Mg alloy obtained within the range of −1.41 V to −1.30 V are plotted in Figure 1B. The shape of the curve in this region was obviously different from that of the three other groups, that is, the current increased over time and no maximum current occurred in the testing process. This phenomenon is related to the overpotential on the electrode surface, and the low overpotential value is unfavorable to metal electrocrystallization (Hsiu et al., 2006). The curves shown in Figure 1C were evidently different from those of the other groups lies because of the “current delay” phenomenon (i.e., the current is almost zero) in the early testing stage. The corresponding delay time gradually decreased as the applied voltage signal increased. In Figure 1D, the response current first reached the maximum value, rapidly decreased, and finally formed the peak current within a relatively short time.

#### 3.1.2 Dimensionless Curves at Different Pulse Signals

**FIGURE 2:**
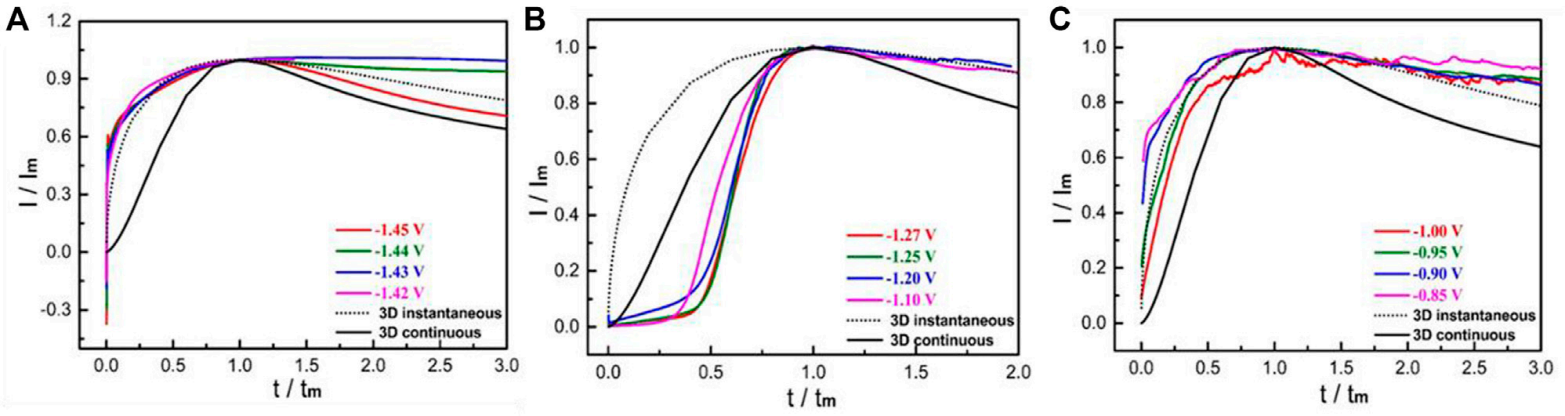
Dimensionless (*I/I*m ∼ *t/t*m) curves at different potential steps: **(A)** −1.42∼−1.45 V; **(B)** −1.10∼−1.27 V; **(C)** −0.85∼−1.00 V.

The current value of two-dimensional nucleation model drops exponentially and decreases to zero, whereas the minimum current of three-dimensional nucleation model tends to be a stable non-zero value with infinite extension of time (Scharifker and Mostany, 1984). Based on the curves in Figure 1, the nucleation model of Mg electrode in the test electrolyte was consistent with the 3D nucleation mechanism. Although there are plenty of expression mathematical models of metal nucleation, the most widely used is dimensionless expression with undetermined parameters in the process of investigating electrocrystallization mechanism by using *i*-*t* transient curves (Wang et al., 2022). The dimensionless processed transient of Mg electrode in the testing solution is displayed in Figure 2. A comparison of the *I/I*_*m*_−*t/t*_*m*_ curves and the three-dimensional theoretical nucleation curves obtained using Equation 1, Equation 2 confirming that it was the three-dimensional instantaneous nucleation model.

### 3.2 Quantitative Analysis of the Nucleation Process of Mg Electrode

According to previous studies, the relationship between *i* ^*1/2*^ and *t* in the 3D instantaneous nucleation equation is linear as follows (Scharifker and Mostany, 1984):

$$i=\frac{\left(ZFK^{\prime }MK^{2}N_{0}t^{2}\right)}{d^{2}},$$ (1)

where *Z* represents the electron transfer number; *F* is the Faraday constant; *N*_*0*_ is the total number of active centers (cm^−2^); *K* is the growth rate of crystals parallel to the matrix; *K′* is the growth rate in the vertical direction (mol^**.**^cm^−2**.**^ s^−1^); and *d* and *m* represent the density (g^**.**^cm^−3^) and the relative molar mass (g^**.**^mol^−1^) of the deposited metal, respectively. By processing the experimental data further, the normal growth constant (*K*) and the mixed growth constant (*K* ^*2*^ *N*_*o*_) at different excitation signals were obtained and the influence of the applied potential on the growth rate and the number of active sites was intuitively determined. In addition, the normal growth rate constant (*K*) linearly related to the maximum current (*i*_*m*_) measured by the transient curve was calculated as follows (Scharifker and Mostany, 1984):

$$K^{\prime }={\text{i}}_{\text{m}}/\left(\text{ZF}\right).$$ (2)

**FIGURE 3:**
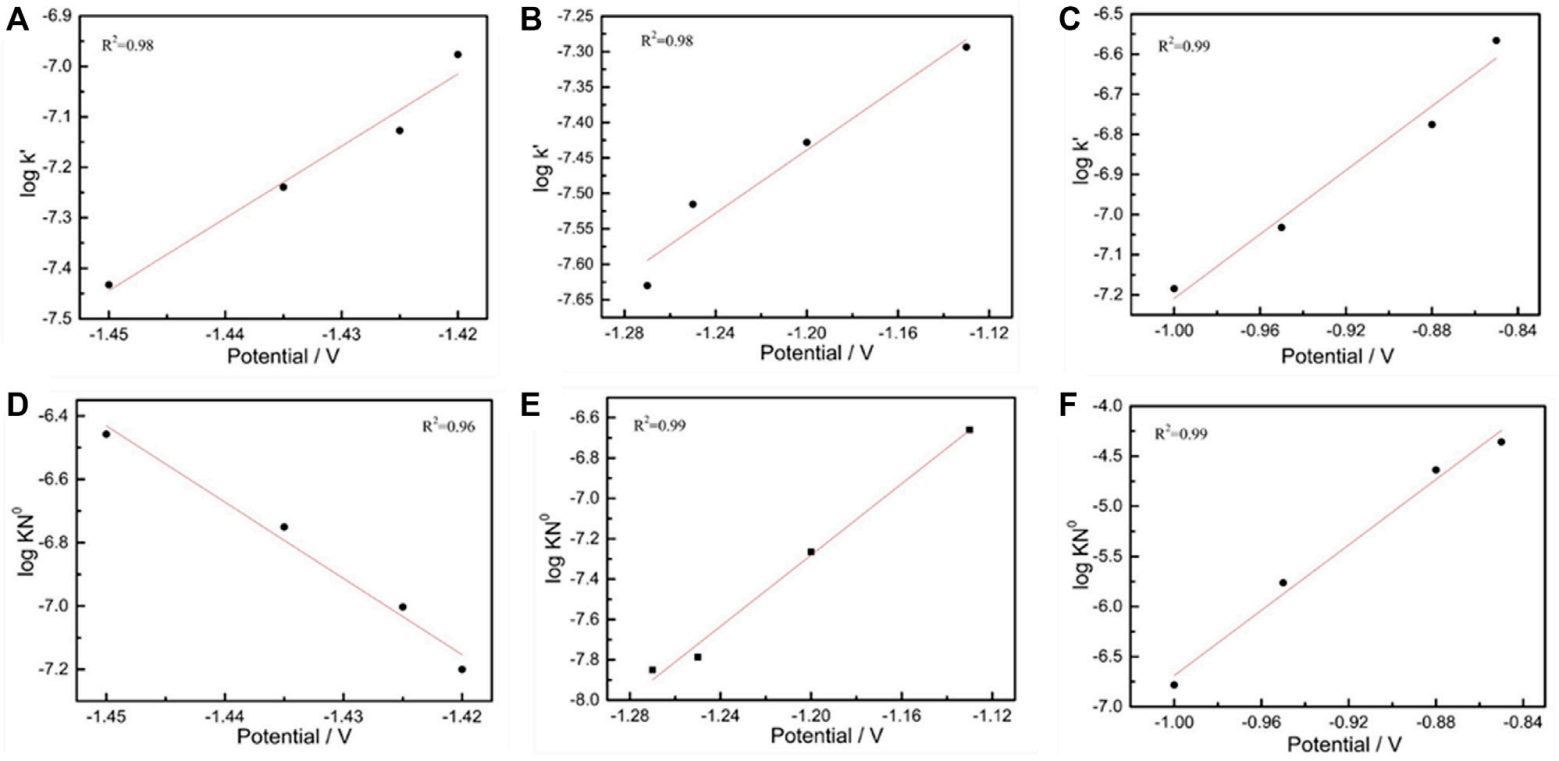
Plot of the logarithm of vertical growth rate constant lg (*K′*) **(A–C)**. Plot of the logarithm of vertical growth rate constant lg (*K* ^2^ *N*_o_) **(D–F)**.

Thus, the corresponding *K′*value was obtained on the basis of the maximum current value by using Equation 2. The value of a logarithm (*lgK*’) was taken, and the applied potential was plotted as the horizontal coordinate (Figure 3). The normal growth constant linearly increased as the applied potential value was increased at all test intervals.

lg *K* ^*2*^ *N*_*0*_ and the applied potential (V) showed a good linear relationship (Figure 3). In the weakly polarized region (−1.40 to −1.30 V), the mixing velocity constant gradually decreased as the potential increased, whereas the two other test intervals displayed the opposite patterns. According to the electrocrystallization theory, a low overpotential can increase the response time of crystal nucleation because of fewer available nucleation sites.

## 4 Conclusion

In summary, the electrochemical results indicated that the transient curves of Mg electrode in aqueous electrolyte follows the three-dimensional instantaneous nucleation model. Qualitative analysis of the current–time curves of the initial nucleation process revealed that the normal growth constant and the mixed growth constant linearly increased with the applied potential signal. This groundbreaking research provides a theoretical basis for the application of MIBs.

## Data Availability

The raw data supporting the conclusions of this article will be made available by the authors, without undue reservation.
